# Sustained AFP-L3 or DCP expression is associated with progression risk and inferior outcomes in unresectable hepatocellular carcinoma

**DOI:** 10.1007/s10238-025-01877-8

**Published:** 2025-10-25

**Authors:** Kelley Núñez, Tyler Sandow, Jeff Burton, Mina Hibino, Juan Gimenez, Ari Cohen, Neil Mehta, Paul Thevenot

**Affiliations:** 1 Institute of Translational Research, Ochsner Health System, 1415 River Road, New Orleans, LA 70121 USA; 2 Interventional Radiology, Ochsner Health System, New Orleans, LA USA; 3 Ochsner Center for Outcomes Research, Ochsner Health System, New Orleans, LA USA; 4 Multi-Organ Transplant Institute, Ochsner Health System, New Orleans, LA USA; 5 Faculty of Medicine, University of Queensland, Brisbane, QLD Australia; 6 Division of Gastroenterology and Hepatology, Department of Medicine, University of California San Francisco, San Francisco, CA USA

**Keywords:** *Lens culinaris* agglutinin fraction of AFP (AFP-L3), Des-gamma-carboxyprothrombin (DCP), Hepatocellular carcinoma (HCC), Liver-directed therapy (LDT)

## Abstract

The HCC biomarkers, *Lens culinaris* agglutinin fraction of AFP (AFP-L3) and des-gamma-carboxyprothrombin (DCP) with AFP, have been approved for surveillance in HCC but have not yet impacted disease management. In this prospective study, AFP, AFP-L3, and DCP were assessed as a non-invasive strategy to monitor residual disease and post-LDT changes in association with risk of disease progression. The biomarkers were measured at HCC diagnosis and following first cycle LDT. Response to LDT was evaluated using mRECIST. Time to event endpoints included time to advanced-stage disease progression, duration of overall complete response (oDoCR), and overall survival. The study included 182 patients with diverse biomarker profiling both at diagnosis and following first cycle LDT. Despite treatment, persistent biomarker expression was found in 36% (66/182) of patients with most (50/66) having elevations in AFP-L3 and/or DCP (AFP-L3^+^/DCP^+^). Overall CR rates were highest in patients treated to triple negative/AFP^+^ only (61%) compared to AFP^+^AFP-L3^+^/DCP^+^ (30%) or AFP_NEG_AFP-L3^+^/DCP^+^ (22%) phenotypes. In patients expressing AFP-L3^+^/DCP^+^ that achieved an overall CR, the oDoCR was lower compared to patients treated to triple negative/AFP^+^. Higher 1-year stage progression rates were also observed in patients with AFP-L3^+^/DCP^+^ phenotypes. Overall survival was lowest in patients expressing AFP^+^AFP-L3^+^/DCP^+^ (2-year rate: 36%). In patients with an incomplete response, triple negative/AFP^+^ phenotypes had longer TTP compared to AFP-L3^+^/DCP^+^ phenotypes (median TTP: 63 months vs 10 months). Sustained AFP-L3^+^/DCP^+^ expression following LDT may be associated with aggressive residual tumor contributing to an incomplete response, increased risk of disease stage progression, and inferior survival outcomes.

**Supplementary Information:**

The online version contains supplementary material available at 10.1007/s10238-025-01877-8.

## Introduction

Hepatocellular carcinoma (HCC) remains the third leading cause of cancer-related deaths worldwide [1] with rising incidence rates due to the increased global prevalence of metabolic dysfunction-associated steatotic liver disease. In early- to intermediate-stage disease, liver-directed therapies (LDTs) have been used as a definitive treatment or bridging/downstage to liver transplantation. A complete radiographic response to LDT significantly delays tumor progression [2] and improves overall survival [3–5] which led to its incorporation in the Barcelona clinic liver cancer (BCLC) staging algorithm. Although treatment options across the staging algorithm have increased, the 5-year survival rate for HCC remains poor at 50% [6] and highlights the opportunity to optimally match aggressive tumor biology to the most effective treatment option to improve overall outcomes.

Alpha-fetoprotein (AFP) remains a key surveillance biomarker for HCC diagnosis and is the main clinical serological indicator of both treatment response and advancing disease [7, 8]. However, AFP-based prognosis has become more challenging in recent years due to improved early detection rates spawned by increased application of and adherence to HCC surveillance programs [9]. In earlier stage disease, 40–50% of patients present with normal AFP levels [10–12]. While these patients express AFP well below established risk thresholds, current evidence suggests a subgroup of patients with aggressive HCC biology and higher disease progression risk in the absence of AFP elevation [2, 13] and suggests an urgent need for additional biomarkers.

Models like GALAD and HES V2.0 have incorporated AFP along with *Lens culinaris* agglutinin fraction of AFP (AFP-L3) and des-gamma-carboxyprothrombin (DCP) for early detection of HCC [14, 15]. While both AFP-L3 and DCP have also been associated with post-surgical recurrence [16], they have yet to achieve clinical translation in the early-intermediate HCC setting. In a large cohort of early-stage HCC patients receiving an array of approved first cycle LDT options, we showed AFP-L3 and DCP expression in combination with AFP was associated with a dismal LDT response and an accelerated 6-month median time to progression resulting in dismal 1-year progression rates [12, 17]. While a multicomponent biomarker profile including AFP-L3 and DCP was associated with inferior first cycle LDT response, the influence of post-treatment biomarker response on downstream prognosis remains unclear. Questions related to whether AFP-L3 and DCP truly identify high-risk patients not captured by AFP alone and how this information could be clinically utilized also remain. In this prospective study of BCLC A -B with unresectable HCC, changes in biomarker profile were assessed following first cycle LDT to evaluate outcomes associated with a biomarker response and as a non-invasive indicator of complete response durability, profile response-based disease progression, and overall survival risk following first cycle LDT.

## Materials and methods

### Study cohort and clinical variables

A prospective, single-center study was conducted in accordance with the ethical guidelines set forth by the 1975 Declaration of Helsinki. The study was approved by the Ochsner Health Institutional Review Board (protocol# 2016.131.B) with written consent obtained from each participant. Enrollment dates spanned from May 2018 to February 2024. Inclusion criteria for the study included: (i) HCC diagnosis confirmed by triple-phase imaging according to the liver imaging-reporting and data system criteria (v2018 American Collect of Radiology) or biopsy confirmed, (ii) unresectable HCC determined by surgical oncologists, (iii) ≥ 18 years of age, and (iv) scheduled to receive first cycle liver-directed therapy (first cycle LDT). Exclusion criteria were (i) warfarin use and (ii) biopsy-confirmed mixed cholangiocarcinoma and HCC, and (iii) missing baseline or post-LDT sample. Clinical variables included: demographics, model of end-stage liver disease scores, complete metabolic liver panels (CMP), and complete blood counts (CBC), and were extracted from the electronic medical record on the date of or within 30 days prior to LDT. Post-treatment model of end-stage liver disease scores, CMP, and CBC were also extracted after follow-up imaging. The time between first cycle LDT and post-treatment laboratory values collected was recorded.

### Liver-directed therapy and response assessment

All liver-directed therapy, including doxorubicin eluting embolic transarterial chemoembolization (DEE-TACE), microwave ablation (MWA), and Yttrium-90 (^90^Y), were performed within a single health care system (Ochsner Health). Institutional criteria to receive LDT included: (i) BCLC-A-B, (ii) ECOG 0–1, and (iii) Child–Pugh score A-B or deemed to have sufficient preserved liver function by the tumor board in consultation with the presenting hepatologist with tumor location and size amendable to LDT. Treatment approach and modality were selected based on the interventional radiology program treatment algorithm factoring in both the size/location of the tumor and the preference of interventional radiology provider.

Response to first cycle LDT was determined by a board-certified interventional radiologist (> 10 years’ experience) using modified response evaluation criteria in solid tumors (mRECIST) [18]. Responses were defined as either overall complete (CR) or incomplete response (IC) which included mRECIST scores of partial, stable, or disease progression. The time between first cycle LDT and assessment of imaging response was recorded.

### Blood collections and biomarker measurements

After informed written consent was obtained, peripheral blood was collected immediately prior to first cycle LDT. A second sample of peripheral blood was collected following first cycle LDT during or near post-treatment imaging. Blood samples were collected in sodium citrate cell preparation tubes (BD Biosciences) and processed within 4 h of collection to obtain plasma. Plasma was immediately stored at -80° C until biomarker measurements. Biomarkers, AFP, AFP-L3, and DCP levels were measured using the μTASWakoi30 (FUJIFILM Wako Diagnostics) on plasma. Minimum detectable ranges for each biomarker were: AFP > 0.30 ng/mL, AFP-L3 > 0.50%, and DCP > 0.10 ng/mL. Biomarker values at the minimum of detection were recorded as the minimum value. Biomarker positivity was determined using previously established thresholds and were AFP > 20 ng/mL, AFP-L3 > 15%, and DCP > 7.5 ng/mL. Time between first cycle LDT and post-treatment biomarker assessment was recorded.

### Study outcomes

The primary study endpoint was time-to-progression (TTP) and was defined as the time of first cycle LDT until stage progression from BCLC stage A or B to BCLC-C defined as the development of extrahepatic metastasis and/or macrovascular invasion following first cycle LDT. Patients were censored for the following: liver transplantation, > 6 months without follow-up or surveillance imaging, all-cause mortality, or free of stage progression to BCLC-C at the time of data analysis. Censoring date was defined as the date from the most recent imaging appointment. Secondary study endpoints were overall survival (OS), overall CR to first cycle LDT as determined by mRECIST, and overall duration of complete response (oDoCR). OS was defined as the time from first cycle LDT until death. oDoCR was determined in only those patients that achieved an overall CR to first cycle LDT and was defined as the time from imaging that confirmed CR to first cycle LDT until either retreatment with LDT, development of new lesion(s), or stage progression to BCLC-C. Patients were censored for the following: liver transplantation, > 6 months without follow-up or surveillance imaging, all-cause mortality, or free of stage progression to BCLC-C at the time of data analysis.

### Statistical analysis

Data analysis was performed using JMP Pro version 18 (SAS Institute Inc.) and SAS version 9.4. All graphical output was generated using GraphPad Prism version 10.0.2 (GraphPad Software Inc.). Categorical variables were listed as a percentage of the total cohort and continuous variables as median with interquartile range (IQR). Logistic regressions analysis was used for factors associated with HCC biomarker groups. Univariate analysis of post-first cycle LDT biomarkers expression associated with TTP was performed using Cox proportional hazards model. Kaplan–Meier curves of TTP, OS, and oDoCR were generated using GraphPad Prism and compared using log-rank tests. Curves were trimmed to the nearest 6-month time point after the at-risk population fell between 10% of the total population being analyzed. The Cox proportional hazards model was used for TTP, with all other events considered censoring events. The fine and gray model for competing risks was used for TTP, with surgical intervention or death unrelated to tumor considered competing events.

## Results

### Study cohort characteristics

**Fig.1:**
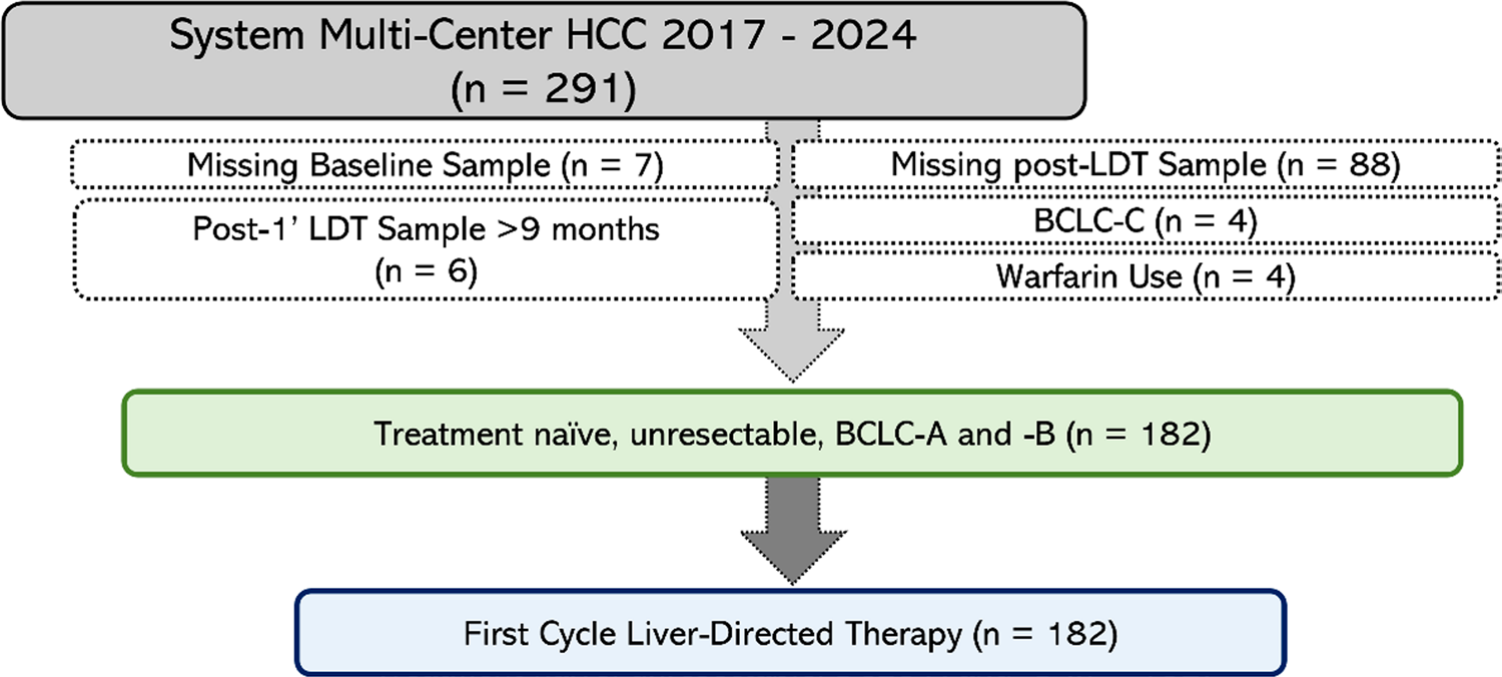
Study consort diagram

The parent observational cohort prospectively followed 291 patients with HCC who received first cycle LDT from 2017–2024. Patients with missing baseline/post-LDT samples or warfarin use were excluded yielding a final cohort size of 182 patients (Fig. 1). Cohort demographics are displayed in Table 1. Briefly, the study cohort was 71% (130/182) male with 71% (130/182) Caucasian, and 55% (101/182) having an underlying liver disease of steatotic liver disease. Majority of patients had solitary HCC (69%, 125/182) and 69% (125/182) with Child–Pugh A score. All patients received first cycle LDT and all were technically successful. For first cycle LDT modality, 68% (124/182) received ^90^Y, followed by 21% (38/182) MWA, and 11% (20/182) DEE-TACE. The median values for baseline biomarkers were 8.3 ng/mL for AFP, 5.2% for AFP-L3, and 2.7 ng/mL for DCP, all below the positive threshold.

**Table 1:** Study cohort demographics and baseline characteristics

Demographic	Cohort
Patients, n (%)	182 (100)
Age at HCC diagnosis, years, median (IQR)	64 (61–68)
Sex, self-reported, male n (%)	130 (71)
Race, self-reported, n (%)	
Caucasian/White	130 (71)
African American/Black	42 (23)
Other	10 (6)
Cirrhotic etiology, n (%)	
SLD	101 (55)
HCV	76 (42)
Other	5 (3)
Scores and Staging	
ECOG Performance Status, n (%)	
Score 0	135 (75)
Score 1	46 (25)
Child–Pugh, n (%)	
A	125 (69)
B	50 (27)
C	7 (4)
Clinical Labs prior to LDT	
Sodium, mM, median (IQR)	139 (137–141)
Creatinine, mg/dL, median (IQR)	0.9 (0.8–1.1)
Bilirubin, mg/dL, median (IQR)	0.9 (0.6–1.5)
Albumin, g/dL, median (IQR)	3.4 (3.0–3.8)
INR, ratio, median (IQR)	1.1 (1.0–1.2)
Platelets, median (IQR)	123 (73–181)
ALBI Grade, n (%)	
Grade 1	32 (18)
Grade 2	128 (70)
Grade 3	22 (12)
HCC Burden and Biomarkers	
BCLC 2022 Stage, n (%)	
A	152 (84)
B	30 (16)
Multifocal, n (%)	
Solitary	125 (69)
Index Lesion Diameter, cm, median (IQR)	3.0 (2.2–4.0)
Cumulative Lesion Size, cm, median (IQR)	3.6 (2.6–5.1)
HCC Biomarkers	
AFP, ng/mL, median (IQR)	8.3 (1.9–52)
AFP-L3, %, median (IQR)	5.2 (0.5–20)
DCP, ng/mL, median (IQR)	2.7 (0.1–14)
Liver-Directed Therapy	
1' LDT Modality, n (%)	
DEE-TACE	20 (11)
MWA	38 (21)
^ 90^Y	124 (68)

Abbreviations: Interquartile range (IQR), Hepatocellular carcinoma (HCC), Hepatitis C virus (HCV), Steatotic liver disease (SLD), Eastern Cooperative Oncology Group (ECOG), Child–Pugh (CP), International normalized ratio (INR), Liver-directed therapy (LDT), Doxorubicin-eluting embolic transarterial chemoembolization (DEE-TACE), Microwave ablation (MWA), Yttrium-90 (90Y), Albumin-Bilirubin (ALBI), Barcelona Clinic Liver Cancer (BCLC), Alpha-fetoprotein (AFP), AFP-Lens culinaris agglutinin (AFP-L3), des-gamma-carboxy prothrombin (DCP)

### Biomarker Profile Diversity in Solitary HCC and Changes Following first cycle LDT

**Fig. 2:**
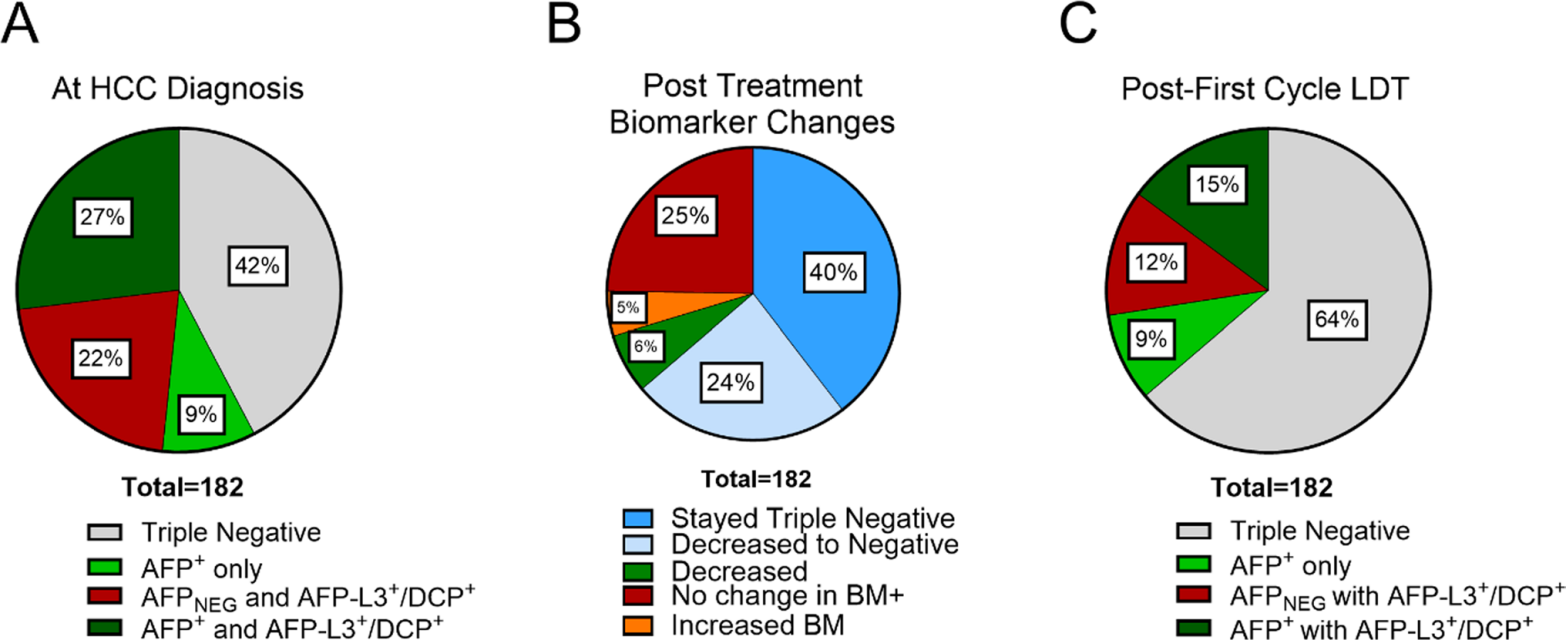
HCC biomarker diversity at diagnosis and following first cycle LDT. **A** Biomarker diversity at HCC diagnosis highlighting the distribution of AFP-L3/DCP expression based on AFP expression. **B** Biomarker changes categorized based on changes in expression profile following LDT. **C** Biomarker diversity following first cycle LDT based on based on AFP expression

Positive biomarker phenotyping at HCC diagnosis revealed diverse AFP-L3 and DCP expression in BCLC A-B disease regardless of AFP expression (Fig. 2A). Only 36% (66/182) of patients had elevated AFP with most (49/66) co-expressing AFP with either AFP-L3 and/or DCP (AFP-L3^+^/DCP^+^) and only a fraction expressed AFP alone (17/66). Biomarker phenotypes were grouped based on positive or negative AFP expression to isolate the subgroup of patients with more complex phenotypes obscured by utilizing AFP alone. This revealed 22% of the cohort were positive for additional biomarkers while maintaining normal AFP levels [AFP_NEG_] (Fig. 2A).

AFP is routinely measured at treatment follow-up as a concordant measure of treatment response and potential indicator of both residual and undetectable disease. Therefore, multi-biomarker profiling was performed at the time of routine imaging follow-up to investigate profile dynamics following first cycle LDT. There was an overall decrease in the number of positive biomarkers following LDT, marked by an 22% increase in the number of triple biomarker negative patients (Fig. 2B). AFP had the highest frequency of biomarker expression (24%) followed by DCP (19%) then AFP-L3 (15%) (Supplemental Table 1). A complete shift in biomarker phenotype from baseline was observed in 37% (68/182) of the cohort following treatment (Supplemental Table 1). Most patients negative for biomarkers prior to treatment, remained negative (72/77), while 24% (44/182) of cohort were treated from positive biomarker to triple negative (Fig. 2B). After treatment, 36% (66/182) of patients remained biomarker positive, with most of this subgroup (68%, 45/66) having no change in biomarker phenotype. While the number of patients with elevated AFP decreased post-LDT, 12% of cohort continued to express AFP-L3 or DCP with normal AFP levels compared to 22% at baseline (Fig. 2C).

### Post-LDT elevations in AFP-L3 and DCP and increased risk of disease progression

**Fig. 3:**
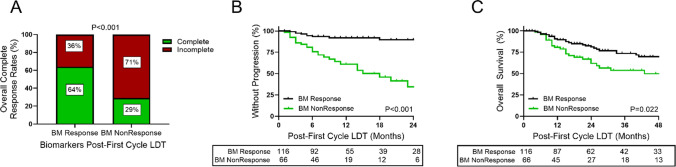
Overall complete response rates, time-to-progression, and overall survival in HCC based on persistent biomarker expression post-treatment. **A** Overall complete response rates of first cycle LDT of patients that stayed or were treated to biomarker negative (BM Response) after LDT versus patients that had persistent biomarker expression (BM NonResponse) after LDT. **B** Patients with persistent biomarker expression (BM NonResponse) were at risk of progression following first cycle LDT. **C** Overall survival was lower in patients that remained biomarker positive (BM NonResponse) following first cycle LDT

Sustained biomarker expression may indicate the presence of residual tumor or radiographically indeterminate but viable HCC. To examine this association, biomarker phenotypes were simplified to a two-component system consisting of biomarker response (defined as stable negative or treatment to negative) or nonresponse (stable positive or detrimental change) (Supplemental Table 2). The overall CR rate in patients that achieved a biomarker response to triple negative was 64% (Fig. 3A). Patients with detrimental or stable biomarker expression had CR rate of 29% (Fig. 3A). Patients with persistent biomarker expression experienced higher rates of post-LDT disease progression compared to patients successfully treated to biomarker negative (1-year rate: 39% vs 8%, 2-year rate: 66% vs 10%) (Fig. 3B). Patients with a biomarker response had longer median TTP compared to patients with persistent biomarker expression (median not reached vs 18 months) which translated to an OS benefit (2-year OS rates: 62% vs 81%; 4-year OS rates: 50% vs 70%) (Fig. 3C).

**Table 2:** Unadjusted and competing risk cox proportional hazards model for TTP

Time interval^a^	Biomarker Group	Reference	Hazard Ratio (95% CI)	
			Model 1: Unadjusted^b^	Model 2: Competing risks^c^
Day	AFP_NEG_AFP-L3^+^/DCP^+^	Triple neg/AFP^+^ only	3.51 (1.31, 9.42)	2.66 (1.04, 6.83)
	AFP^+^AFP-L3^+^/DCP^+^	Triple neg/AFP^+^ only	12.78 (6.39, 25.60)	10.75 (5.43, 21.31)
	AFP^+^AFP-L3^+^/DCP^+^	AFP_NEG_AFP-L3^+^/DCP^+^	3.65 (1.43, 9.32)	4.04 (1.55, 10.52)

^a^Survival assessed daily following liver-directed therapy date

^b^Model 1: Cox proportional hazards model with competing risk events treated as censoring events. Explanatory variable/predictor for biomarker group included only post-treatment biomarker group

^c^Model 2: Fine-Gray model for competing risks. Explanatory variable/predictor for biomarker group included only post-treatment biomarker group

Both (1) patients for whom no event has yet occurred and (2) patients for whom the event of interest has not yet occurred but a competing risk event has previously occurred are considered when estimating hazard functions and ratios. Fine-Gray models can be used to estimate the cumulative incidence function (CIF), providing estimated probabilities of the event of interest over time *in the presence of competing risks*

Abbreviations: *CI* confidence interval, *CR* competing risks

**Fig. 4:**
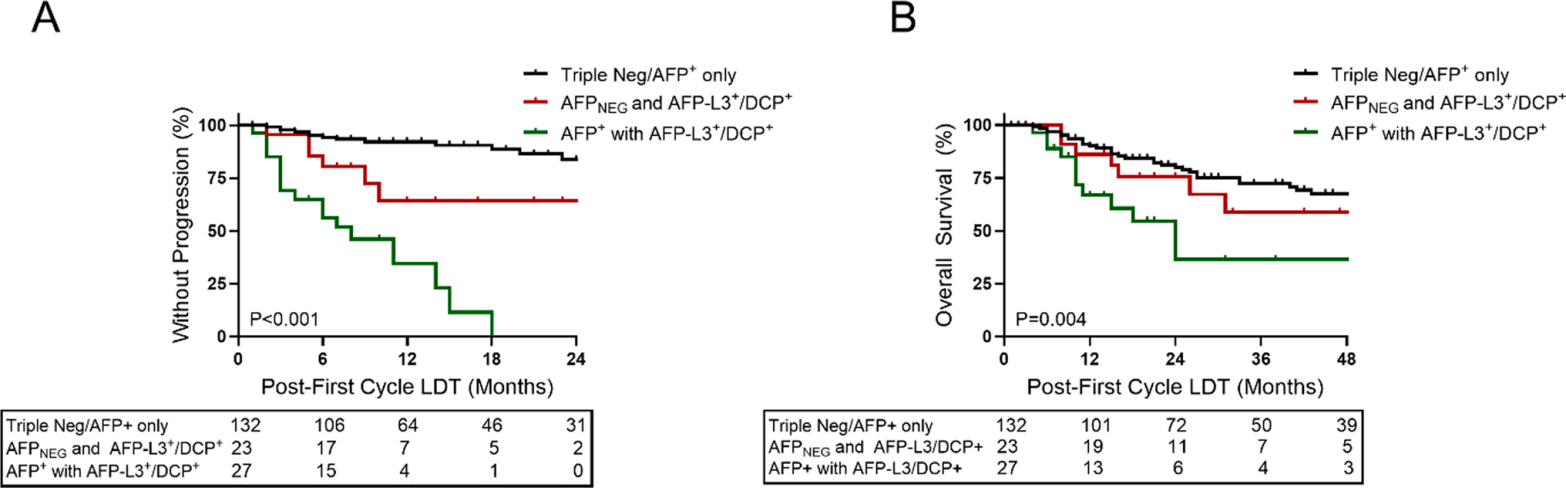
Time-to-progression and overall survival in HCC based on post-liver-directed therapy biomarker expression profiles. **A** Patients expressing additional HCC biomarkers, AFP-L3 or DCP with or without AFP, were at risk of progression following first cycle LDT. **B** Overall survival based on biomarker profiles following LDT

While biomarker response can stratify outcomes within treatment response subgroups, this approach assumes that AFP-L3 and DCP were clinically available to inform patient care. To isolate and evaluate the potential added benefit of including AFP-L3 and DCP with AFP, biomarker phenotypes were reevaluated by anchoring to AFP expression and isolating the AFP-L3^+^/DCP^+^-containing phenotypes. Positive expression of any individual biomarker following LDT was associated with an increased risk of disease progression (Supplemental Table 3). Analysis of biomarker accumulation demonstrated an optimal hazard ratio demarcation between 0–1^+^ versus 2–3^+^ biomarkers, supporting increased progression risk associated with multi-biomarker phenotypes. AFP-L3^+^/DCP^+^ phenotypes were isolated to produce subgroups that included triple negative/AFP positive, AFP_NEG_ and AFP-L3^+^/DCP^+^, and AFP^+^ with AFP-L3^+^/DCP^+^. Patients with elevations in AFP-L3 or DCP had increased risk of disease progression within 1-year following first cycle LDT (Fig. 4A). Progression risk increased in multiple positive profiles that co-expressed AFP-L3^+^/DCP^+^ with elevated AFP compared to those expressing AFP-L3^+^/DCP^+^ with normal AFP (1-year rates 65% vs 36%). Patients treated to triple biomarker negative or expressing AFP alone had a low 1-year progression rate of 8% following LDT. Hazard ratios for the AFP^+^ with AFP-L3^+^/DCP^+^ group was 12.8 (95% CI 6.4–25.6), once accounting for competing risks, hazard ratios were 10.8 (95% CI 5.4–21.3) (Table 2). Post-LDT profiles expressing AFP^+^ with AFP-L3^+^/DCP^+^ also yielded inferior OS outcomes compared to AFP-L3^+^/DCP^+^ with normal AFP or the biomarker negative/AFP alone subgroup (2-year OS rate: 36% vs 76% and 80%) (Fig. 4B).

**Table 3:** Unadjusted and competing risk cox proportional hazards model for duration of complete response

Outcome^a^	Biomarker group	Reference	Hazard Ratio (95% CI)	
			Model 1: Unadjusted^b^	Model 2: Competing risks^d^
oDoCR	AFP (+ / −) w/AFP-L3^+^/DCP^+^	Triple neg/AFP^+^ only	3.81 (1.20, 12.10)	2.81 (0.89, 8.87)

^a^Survival assessed daily from imaging date confirming complete response

^b^Model 1: Cox proportional hazards model with competing risk events treated as censoring events. Explanatory variable/predictor for biomarker group included only post-treatment biomarker group

^c^Model 3: Fine-Gray model for competing risks. Explanatory variable/predictor for biomarker group included only post-treatment biomarker group

Both (1) patients for whom no event has yet occurred and (2) patients for whom the event of interest has not yet occurred but a competing risk event has previously occurred are considered when estimating hazard functions and ratios. Fine-Gray models can be used to estimate the cumulative incidence function (CIF), providing estimated probabilities of the event of interest over time *in the presence of competing risks*

Abbreviations: *CI* confidence interval, *CR* competing risks, *oDoCR* overall duration of complete response

### Elevations in AFP-L3 and DCP and response to first cycle LDT

While a numerical increase in biomarkers has been inversely correlated with LDT objective response rate, it is unclear whether inferior response rates are intimately associated sustained AFP-L3^+^/DCP^+^ expression. A breakdown of demographic and baseline factors isolating the post-LDT AFP-L3^+^/DCP^+^-containing phenotypes from the triple negative/AFP alone group revealed the more complex biomarker phenotypes contained a higher percentage of Child–Pugh B-C underlying disease (39–44% compared to 27%) and a larger median tumor size of 3.8 cm which contributed to an increase in BCLC-B staging due to multifocal disease (Supplemental Table 4). However, albumin and bilirubin levels were well-controlled across the phenotypes and there was no difference in LDT approach despite the 1 cm increase in index tumor size. The median time to follow-up biomarker assessment was also well controlled across phenotypes.

**Fig. 5:**
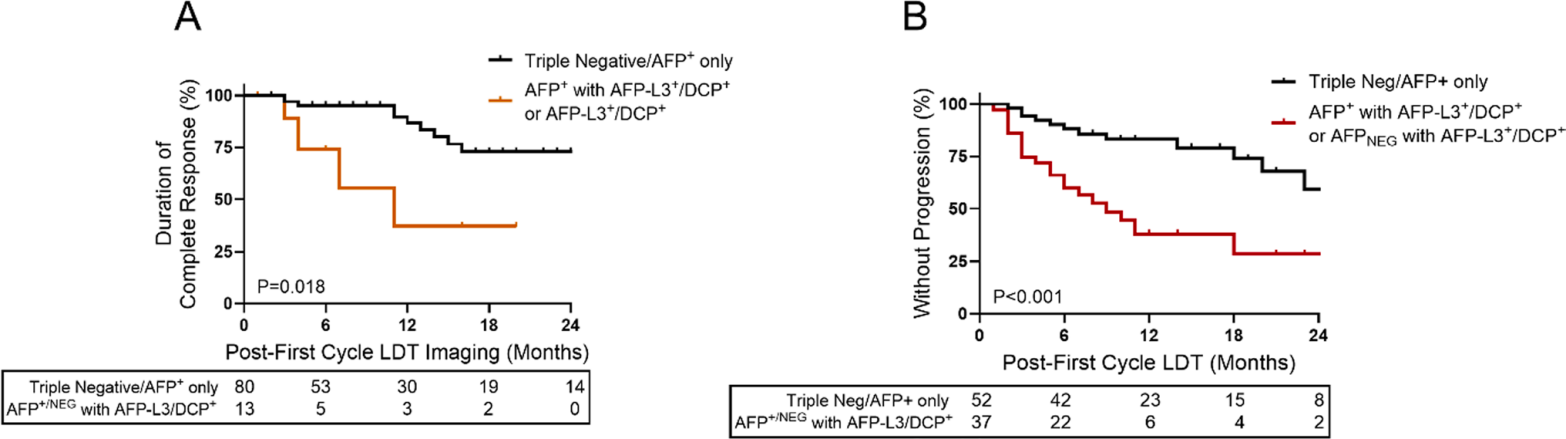
Time-to-progression in patients with incomplete response and duration of complete response based on post-liver-directed therapy biomarker expression. **A** Duration of complete response in patients who achieved a complete radiographic response to first cycle LDT, based on post-treatment biomarker profiles. **B** TTP in patients who had an incomplete response to first cycle LDT based on post-treatment biomarker profile

Post-LDT profiles consisting of triple negative/AFP alone had superior treatment response rates, with an overall CR rate of 61% compared to 22% in AFP_NEG_ AFP-L3^+^/DCP^+^ and 30% in AFP^+^ with AFP-L3 + /DCP + . While only a fraction of patients expressing AFP-L3^+^/DCP^+^ achieved an overall CR (13/93), the duration of CR was short-lived with 44% requiring target retreatment by the second 3-month surveillance cycle (Fig. 5A). Hazard ratios for the AFP-L3^+^/DCP^+^ group were 3.8 (95% CI 1.2–12.1), once accounting for competing risks, hazard ratios were 2.8 (95% CI 0.89–8.9) (Table 3). Conversely, only 14% of patients with a triple negative or AFP alone profile after CR to LDT required target retreatment within the 1-year of initial treatment. In patients with an initial incomplete response requiring immediate retreatment, patient expressing triple negative or AFP alone were less likely to experience disease progression within the first following initial treatment, with a 17% progression rate at 1-year compared to 62% in the AFP-L3^+^/DCP^+^ group (Fig. 5B). Median TTP for AFP-L3^+^/DCP^+^ was 9 months compared to 63 months in triple negative or AFP only phenotype.

## Discussion

Recent studies have continued to show a decline in the percentage of patients with dramatic AFP elevation at early-stage diagnosis and, in parallel, have shown overall declining AFP levels approaching the upper limit of normal [19]. This cohort aligns with these trends, revealing only 36% of patients with a recent diagnosis of HCC were AFP^+^ with only 24% remaining positive after first cycle LDT. In the absence of AFP elevation, critical aspects of disease management utilizing AFP must solely rely on radiographic indicators, including treatment response concordance [20], as a non-invasive indicator of local recurrence or advancing disease [20, 21], and to identify surgical candidates with lower recurrence risk [22]. This increases our reliance on post-treatment triple-phase imaging, where postsurgical data have consistently shown a high discordance between complete imaging response and complete pathologic response [23]. And while imaging response, particularly first cycle response, is associated with HCC outcomes, roughly 30% either do not initially respond or fail to sustain an apparent CR through the first imaging surveillance cycle [23]. Our understanding of the factors associated with sustaining a CR are limited to AFP response in patients initially expressing AFP. Further, the inability to identify non-responders prior to treatment restricts our ability to trial optimal first cycle treatment pathways in patients at high-risk of rapid progression beyond BCLC A-B.

The initial success [24] and projected increased utilization of the GALAD score for HCC surveillance has and will continue to increase the clinical availability of AFP, AFP-L3, and DCP profile data at the time of diagnosis. We and others have shown that multi-biomarker profiling provides critical insight into HCC biological aggressiveness that is independent of underlying etiology of liver disease, progression of cirrhosis, or radiographic tumor burden [12, 16, 17, 25–27]. Additional work, using a variety of biomarker cutoffs with a wide range of positive threshold levels, such as DCP levels up to tenfold lower than those recommended for HCC surveillance, have focused on OS [28, 29], but have yet to advocate their use to drive optimal staging and definitive treatment plans. The biomarker thresholds in this analysis are anchored in the current recommendations for HCC surveillance and utilizing data available at the time of diagnosis, providing a direct translatable path to clinical disease management. Profiling can stratify treatment response outcomes and tumor progression risk in BCLC A-B patients who primarily receive LDT as a first line of treatment and is also effective in stratifying outcomes discreetly within BCLC-A or BCLC-B. We recently showed that biomarker profiling can identify treatment non-responder subgroups in otherwise optimal BCLC-A disease, suggesting profiling may have a future role in modifying disease staging and candidacy for advanced treatment approaches including combination systemic therapy.

In agreement with multi-biomarker data from other HCC cohorts, we find complex multi-biomarker expression profiles, despite a BCLC A-B diagnosis, with most positive profiles containing AFP-L3^+^/DCP^+^. Even more concerning, many patients continued to express these high-risk biomarkers following LDT (25% of cohort, 46/182), where they may provide a valuable early indication of residual disease, viable indeterminate disease, and impending risk of disease progression and could be leveraged to accelerate treatment timelines or transition to more aggressive treatment options. The existing literature on AFP dynamics following LDT suggests a relationship between AFP response and LDT response while providing prognosis for HCC progression risk and overall survival [20]. However, similar response evidence with AFP-L3 and DCP has been limited to pre-surgical analysis and biased to patients who have optimally responded to LDT [16, 30]. In this cohort, patients with sustained AFP-L3^+^/DCP^+^ expression following LDT, regardless of AFP co-expression, were at significantly higher risk of disease progression, particularly within the first year of treatment. Longitudinal biomarker response data could potentially impact both treatment approach and treatment options, particularly in patients where liver transplantation was initially the definitive treatment approach. By extension, this could also have treatment target implications bridging or downstaging patients given the minimal 2-year risk of disease progression in patients treated to triple negative or with mild residual AFP expression following initial LDT treatment plan.

Sustained, and specifically, increased AFP expression in patients with an apparent CR may trigger a thorough tumor board review and include transarterial angiography to identify other sources of tumor-feeding vessels or investigate indeterminate observations. Given the dismal implications of sustained AFP-L3 and/or DCP identified in this study, AFP-L3^+^/DCP^+^ expression in the setting of a radiographic CR may warrant further investigation through interventional radiology as only a fraction of these maintained an overall CR beyond the first surveillance cycle. This data continues to suggest that not only is baseline AFP-L3^+^/DCP^+^ expression associated with more aggressive HCC biology, but also that sustained expression may be a clear indicator of aggressive residual disease warranting immediate retreatment. The developing link between AFP-L3^+^/DCP^+^ and biologically aggressive, viable HCC are aligned with pre-surgical multi-biomarker liver transplant studies linking expression with the presence of viable tumor with aggressive biological features and the risk of post-transplant disease recurrence [16, 25–27].

The inferior outcomes with sustained AFP-L3^+^/DCP^+^ expression were independent of underlying etiology of liver disease and serological markers of liver function while also notably controlled for time to biomarker follow-up. While AFP-L3^+^/DCP^+^ expressing patients were more likely to have larger index size (2.8 cm vs 3.8 cm), the cumulative tumor burden and LDT approach were similar between phenotypes suggesting a similar clinical approach to treatment. Accumulating data continue to suggest AFP, AFP-L3, DCP profiling provides a patient-specific and tumor-direct measure of biological aggressiveness with prognostic implications for short-term risk of progression to advance-stage disease. In this study, BCLC A-B patients expressing AFP with AFP-L3^+^/DCP^+^ at diagnosis had 2-year OS rates of 36%, similar to patient outcomes associated with BCLC-C stage disease. Results from combined LDT with immune checkpoint inhibitor clinical trials (EMERALD-01 and LEAP-012) may provide an alternative treatment pathway for BCLC A-B patients with aggressive biomarker profiles and a high-risk of progression with LDT alone.

At the other end of spectrum, we found that patients with only sustained AFP expression had similar first cycle response rates and time to event outcomes as biomarker triple negative patients. While the literature has well established AFP risk thresholds, most are well above the highest quartiles for recent early-stage HCC cohorts at 400 ng/mL to as high as 1000 ng/mL and more likely indicative of vascular invasion and/or metastasis. However, in early-stage disease, lower AFP thresholds < 100 ng/mL have been linked to treatment response, local recurrence risk, and overall progression risk. While the population of patients expressing only AFP in our cohort is limited, it is plausible that a portion of the historical risk associated with AFP expression could be attributed AFP-L3^+^/DCP^+^ co-expression. The collective AFP-L3/DCP data in BCLC A-B disease continues to support a clinical benefit to utilizing the panel to inform disease management processes.

The study was designed as a retrospective analysis of prospectively collected data with a consistent methodology and protocol across the study timeline. Although the study has limitations related to the single center design, concerns of bias related to regional variables or methodology may be unfounded based on the similar findings in presurgical patients in other recent single center studies [16, 25–27]. An additional assay-specific limitation to the AFP, AFP-L3, and DCP system is the DCP false positive rate encountered in patients taking the anticoagulant warfarin, although only a small percentage of patients met this exclusion criteria in this cohort.

## Conclusion

In conclusion, with limited HCC molecular and genetic data in BCLC A-B disease, non-invasive biomarkers associated with aggressive tumor biology are in urgent need. The AFP, AFP-L3, DCP system in conjunction with radiographic imaging provides an ideal platform to assess baseline prognosis and treatment response while also identifying patients at high-risk for aggressive residual disease or local recurrence. In combination, the biomarker panel provides an effective treatment target for durable disease control and may help identify treatment responder populations in patients with more advanced disease. While initial and sustained AFP with AFP-L3^+^/DCP^+^ was associated with high progression risk in patients treated with LDT, a potential benefit from combination of LDT and immunotherapy warrants future investigation.

## Supplementary Information

Below is the link to the electronic supplementary material.

Supplementary file1 (PDF 151 kb)

## Data Availability

All data included in this study are available upon reasonable request to the corresponding author.

## Abbreviations

HCC: Hepatocellular carcinoma
LDT: Liver-directed therapy
BCLC: Barcelona clinic liver cancer
AFP: Alpha-fetoprotein
AFP-L3: *Lens culinaris* Agglutinin fraction of AFP
DCP: Des-gamma-carboxyprothrombin
CMP: Complete metabolic panel
CBC: Complete blood counts
DEE-TACE: Doxorubin-eluting embolic transarterial chemoembolization
MWA: Microwave ablation
^90^Y: Yttrium-90
CR: Complete response
IC: Incomplete response
mRECIST: Modified response evaluation criteria in solid tumors
TTP: Time-to-progression
OS: Overall survival
oDoCR: Overall duration of complete response
