# Supplementary material for: Sustained AFP-L3 or DCP expression is associated with progression risk and inferior outcomes in unresectable hepatocellular carcinoma
Source: Clin Exp Med. 2025 Oct 25;25(1):328. doi: 10.1007/s10238-025-01877-8 (PMC12553611; doi:10.1007/s10238-025-01877-8)
Supplement: Supplementary file 1 — Supplementary file1 (PDF 151 kb) [file 10238_2025_1877_MOESM1_ESM.pdf]

SUPPLEMENTAL TABLES

**Supplemental Table 1. Post-Treatment Biomarker Profile and Changes in Expression Profile**	
**HCC Biomarkers**	
AFP, ng/mL, median (IQR)	7.5 (0.3 – 17)
AFP-L3, %, median (IQR)	0.5 (0.5 – 0.5)
DCP, ng/mL, median (IQR)	0.1 (0.1 – 4.1)
AFP positive, n (%)	43 (24)
AFP-L3 positive, n (%)	28 (15)
DCP positive, n (%)	35 (19)
**Biomarker Groups**	
**Stayed Triple Negative, n (% of total)**	72 (40%)
Remained Triple Negative	72
**Decreased to Negative, n (% of total)**	44 (24%)
AFP+ → Triple Negative	6
AFP-L3+ → Triple Negative	7
DCP+ → Triple Negative	16
AFP+ and AFP-L3+ → Triple Negative	9
AFP+ and DCP+ → Triple Negative	1
AFP-L3+ and DCP+ → Triple Negative	1
Triple Positive → Triple Negative	4
**Decreased Biomarker+, n (% of total)**	12 (6%)
AFP+ and AFP-L3+ → AFP-L3+	3
AFP-L3+ and DCP+ → DCP+	1
AFP+ and DCP+ → AFP+	2
AFP+ and DCP+ → DCP+	2
Triple Positive → AFP+	1
Triple Positive → AFP+ and AFP-L3+	1
Triple Positive → AFP+ and DCP+	1
Triple Positive → DCP+	1
**Increased Biomarker+, n (% of total)**	9 (5%)
Triple Negative → DCP+	4
Triple Negative → AFP+	1
AFP+ and AFP-L3+ → Triple Positive	1
AFP+ and DCP+ → Triple Positive	1
DCP+ → AFP+ and DCP+	1
AFP-L3+ → AFP-L3+ and DCP+	1
**No Change in Biomarker+, n (% of total)**	45 (25%)
Remained Triple Positive	10
Remained AFP+ and AFP-L3+	10
Remained AFP+ and DCP+	1
Remained AFP+	11
Remained DCP+	10

AFP+ and DCP+ → AFP+ and AFP-L3+	1
DCP+ → AFP+	1
AFP-L3+ → DCP+	1
**Abbreviations: Liver-directed therapy (LDT), Alpha-fetoprotein (AFP), AFP-*Lens culinaris* agglutinin (AFP-L3), des-gamma-carboxy prothrombin (DCP).**	

Supplemental Table 2. Cox Proportional Hazards Analysis of Biomarker Response Levels Associated with Time to Progression

Biomarkers Changes Grouped	P value	HR (95% CI)
Decreased to Negative vs Stayed Triple Negative	0.679	
Increased BM+ vs Decreased BM+	**0.020**	7.1 (1.4 – 37)
No Change BM+ vs Decreased BM+	0.068	
No Change BM+ vs Increased BM+	0.238	
No Change BM+ vs Stayed Triple Negative	**<0.001**	7.2 (2.9 – 18)
Biomarker Response (BM^{NEG}) vs No Response (BM+)	**<0.001**	5.6 (2.8 – 12)

Abbreviations: Biomarker (BM), Negative (NEG).

Supplemental Table 3. Cox Proportional Hazards Analysis of Biomarker Expression and Profile Phenotypes Associated with Time to Progression

**Positive Biomarkers Post 1' LDT**	**P value**	**HR (95% CI)**
AFP, > vs < 20 ng/mL	<0.001	4.9 (2.5 – 9.3)
AFP-L3, > vs < 15%	<0.001	7.7 (3.8 – 15.3)
DCP, < vs > 7.5 ng/mL	<0.001	5.8 (3.0 – 11.4)
Positive Biomarkers, 0-3	<0.001	
1 vs 0	0.022	2.8 (1.2 – 6.8)
2 vs 0	<0.001	10.9 (4.0 – 29)
3 vs 0	<0.001	20 (7.9 – 50)
2 vs 1	0.009	3.9 (1.4 – 11)
3 vs 1	<0.001	7.1 (2.8 – 18)
3 vs 2	0.220	
**Biomarker Groups**	<0.001	
AFP^{+} only vs Triple Negative	0.152	
AFP$_{NEG}$ and AFP-L3^{+}/DCP^{+} vs Triple Negative	0.007	4.0 (1.5 - 11)
AFP^{+} with AFP-L3^{+}/DCP^{+} vs Triple Negative	<0.001	14 (6.1 – 32)
AFP^{NEG} and AFP-L3^{+}/DCP^{+} vs AFP^{+} only	0.394	
AFP^{+} with AFP-L3^{+}/DCP^{+} vs AFP^{+} only	0.002	6.0 (1.9 – 19)
AFP^{+} with AFP-L3^{+}/DCP^{+} vs AFP$_{NEG}$ and AFP-L3^{+}/DCP^{+}	0.011	3.5 (1.3 – 9)

Abbreviations:Liver-directed therapy (LDT), Alpha-fetoprotein (AFP), AFP-*Lens culinaris* agglutinin (AFP-L3), des-gamma-carboxy prothrombin (DCP).

Supplemental Table 4. Demographics and Characteristics of the Study Cohort Based on Post-LDT Biomarker Profile.

Demographic	Triple negative or AFP+ only	AFP_{NEG} and AFP-L3^{+}/DCP+	AFP+ with AFP-L3^{+}/DCP+	P value
**Patients, n (%)**	132	23	27	
**Age at HCC diagnosis, years, median (IQR)**	65 (61 – 68)	64 (60 – 67)	64 (60 – 67)	0.717
**Sex, self-reported, male n (%)**	94 (71)	17 (74)	19 (70)	0.957
**Race, self-reported, n (%)**				0.288
Caucasian/White	92 (70)	19 (83)	19 (70)	
African American/Black	33 (25)	4 (17)	5 (19)	
Other	7 (5)	0 (0)	3 (11)	
**Cirrhotic etiology, n (%)**				0.171
SLD	79 (60)	12 (52)	10 (37)	
HCV	49 (37)	11 (48)	16 (59)	
Other	4 (3)	0 (0)	1 (4)	
**Scores and Staging**				
**ECOG Performance Status, n (%)**				0.736
Score 0	99 (75)	15 (68)	21 (78)	
Score 1	33 (33)	7 (32)	6 (220	
**Child-Pugh, n (%)**				**0.011**
A	96 (73)	14 (61)	15 (56)	
B	33 (25)	5 (22)	12 (44)	
C	3 (2)	4 (17)	0 (0)	
**Clinical Labs after First Cycle LDT**				
**Sodium, mM, median (IQR)**	139 (137 – 140)	138 (135 – 139)	138 (135 – 139)	0.558
**Creatinine, mg/dL, median (IQR)**	0.9 (0.8 – 1.1)	1.0 (0.8 – 1.2)	0.9 (0.8 – 1.0)	0.071
**Bilirubin, mg/dL, median (IQR)**	0.8 (0.6 – 1.5)	1.2 (0.7 – 1.9)	1.2 (0.7 – 1.8)	0.064
**Albumin, g/dL, median (IQR)**	3.4 (3.0 – 3.7)	3.2 (2.8 – 3.6)	3.2 (2.8 – 3.5)	0.116
**INR, ratio, median (IQR)**	1.1 (1.0 – 1.2)	1.1 (1.0 – 1.2)	1.1 (1.0 – 1.2)	0.967
**Platelets, median (IQR)**	120 (78 – 196)	119 (81 – 187)	118 (81 – 190)	0.792

**MELD 3.0, score (IQR)**	9 (7 – 12)	10 (8 – 17)	10 (8 – 15)	0.059
**Time from First Cycle LDT to laboratory, months, median (IQR)**	2.0 (1.1 – 3.5)	1.9 (1.1 – 3.2)	1.6 (1.1 – 3.4)	0.715
**HCC Burden**				
**Index Lesion Diameter, cm, median (IQR)**	2.8 (2.2 – 3.7)	3.8 (2.7 – 4.7)	3.8 (2.7 – 4.9)	**0.013**
**Cumulative Lesion Size, cm, median (IQR)**	3.5 (2.4 – 4.6)	4.5 (3.3 – 6.3)	4.4 (2.8 – 6.7)	0.064
**BCLC Staging**				**0.031**
A	116 (88)	18 (78)	18 (67)	
B	16 (12)	5 (22)	9 (33)	
**Solitary Burden, n (%)**	93 (70)	16 (70)	16 (59)	0.531
**Time from First Cycle LDT to Biomarker assessment, months, median (IQR)**	1.6 (1.0 – 3.0)	2.1 (1.1 – 3.2)	1.6 (1.1 – 3.7)	0.472
**Liver-Directed Therapy**				
**First Cycle LDT Modality, n (%)**				0.925
DEE-TACE	16 (12)	2 (9)	2 (7)	
MWA	28 (21)	5 (22)	5 (19)	
^{90}Y	88 (67)	16 (69)	20 (74)	
**Overall Response to First Cycle LDT**				**<0.001**
Complete response, n (%)	80 (61)	5 (22)	8 (30)	
Incomplete response, n (%)	52 (39)	18 (78)	19 (70)	
**Time from First Cycle LDT to imaging response, months, median (IQR)**	1.6 (1.1 – 3.0)	1.3 (1.1 – 2.8)	2.4 (1.4 – 3.1)	0.672

Abbreviations: Interquartile range (IQR), Hepatocellular carcinoma (HCC), Hepatitis C virus (HCV), Steatotic liver disease (SLD), Eastern Cooperative Oncology Group (ECOG), Child-Pugh (CP), International normalized ratio (INR), Liver-directed therapy (LDT), Doxorubicin-eluting embolic transarterial chemoembolization (DEE-TACE), Microwave ablation (MWA), Yttrium-90 (90Y),Model End-Stage Liver Disease (MELD), Barcelona Clinic Liver Cancer (BCLC), Alpha-fetoprotein (AFP), AFP-*Lens culinaris* agglutinin (AFP-L3), des-gamma-carboxy prothrombin (DCP).
